# Statement by the Commission for Infection Prevention and Hygiene in Healthcare and Nursing (KRINKO) on the indication-appropriate use of mouth-nose protection (MNS) in healthcare

**DOI:** 10.3205/dgkh000628

**Published:** 2026-02-17

**Authors:** Commission for Infection Prevention and Hygiene in Healthcare and Nursing (KRINKO)

**Affiliations:** 1 Robert Koch Institute, Berlin, Germany

**Keywords:** medical face masks, sustainability, indications, misindications, IPC

## Abstract

**Introduction:**

When using a mouth-nose protection (MNS), the indications for their use are not always clear in practice, so that they are often worn even if it is not indicated for infection prevention and control (IPC). This affects the ecological balance and use of resources.

**Method:**

Indications and misindications for the use of MNS were extracted from KRINKO recommendations and other publications and presented in a structured manner.

**Measures and strategies:**

Three types of MNS which are distinguished in the DIN EN 14683 are presented. Examples for typical indications for medical face masks for healthcare workers, patients and external persons are summarized. Examples of situations in which the use of medical face masks is not indicated are also shown. By reducing the use of medical face masks through strictly indication-based use, clarification of the areas of application for the three types of MNS, and differentiated and indication-based use of FFP2 masks, a responsible and sustainable use of medical face masks can be implemented in accordance with IPC measures.

## Zusammenfassung

**Einleitung:**

Bei der Verwendung eines medizinischen Mund-Nasen-Schutzes (MNS) sind die Indikationen für dessen Einsatz im Gesundheitswesen nicht immer klar, sodass MNS häufig auch dann getragen werden, wenn dies nicht als Maßnahme zur Infektionsprävention und -kontrolle (IPC) angezeigt ist. Dies hat Auswirkungen auf die Ökobilanz und den Ressourceneinsatz.

**Methode:**

Indikationen und Kontraindikationen für die Verwendung von MNS wurden aus den Empfehlungen der KRINKO und aus anderen Veröffentlichungen extrahiert und strukturiert dargestellt.

**Maßnahmen und Strategien:**

Es werden die drei MNS-Typen nach DIN EN 14683 vorgestellt. Beispiele für typische Indikationen für MNS für Beschäftigte im Gesundheitswesen und für Patienten werden zusammengefasst. Beispiele für Situationen, in denen die Verwendung von MNS nicht angezeigt ist, werden ebenfalls aufgeführt. Durch eine streng indikationsbasierte Verwendung von MNS, die Präzisierung des Einsatzes der drei MNS-Typen und die differenzierte Verwendung von FFP2-Masken unter Berücksichtigung der gegebenen Indikationen kann eine verantwortungsvolle Verwendung von MNS im Einklang mit IPC-Maßnahmen erreicht werden.

## Table of contents

1 Introduction, reason and objective

2 Definitions

3 Infection prevention/hygiene background

4 Infection prevention/hygienic measures and strategies

4.1 Reducing the use of MNS through strictly indication-based use

4.2 Clarification of the areas of application for the three types of MNS defined in DIN EN 14683

4.3 Avoiding the inappropriate use of FFP2 masks

5 Outlook

Conflict of interest

Literature

### Legal notice

**The content of this statement is intended solely to inform the public, professional circles, or the highest state health authorities and does not constitute a recommendation by KRINKO within the meaning of Sections 23 and 35 (1) of the Infection Protection Act (IfSG).**

This translation is intended solely to provide information to the interested, non-German-reading public. Any discrepancies or differences that may arise in translation of the official German version „Stellungnahme der Kommission für Infektionsprävention in medizinischen Einrichtungen und in Einrichtungen und Unternehmen der Pflege und Eingliederungshilfe (KRINKO) beim Robert Koch-Institut (RKI): Indikationsgerechte Verwendung eines medizinischen Mund-Nasen-Schutzes (MNS) im Gesundheitswesen“ (Epid Bull 2026;1:3-9, DOI: https://doi.org/10.25646/13571) are not binding and have no legal effect.

### Legal notice in German

#### Rechtlicher Hinweis

**Die Ausführungen in dieser Stellungnahme dienen ausschließlich zur Unterrichtung der Öffentlichkeit, von Fachkreisen oder der obersten Landesgesundheitsbehörden und sind keine Empfehlung der KRINKO im Sinne der §§ 23, 35 Absatz 1 Infektionsschutzgesetz (IfSG).**

Rechtlich bindend ist die deutsche Originalfassung Stellungnahme der Kommission für Infektionsprävention in medizinischen Einrichtungen und in Einrichtungen und Unternehmen der Pflege und Eingliederungshilfe (KRINKO) beim Robert Koch-Institut (RKI): Indikationsgerechte Verwendung eines medizinischen Mund-Nasen-Schutzes (MNS) im Gesundheitswesen“ (Epid Bull 2026;1:3-9, DOI: https://doi.org/10.25646/13571). Die englische Fassung dient der Information der internationalen Fachöffentlichkeit.

## 1 Introduction, reason and objective

**Healthcare facilities contribute significantly to global greenhouse gas emissions **[1]** and are responsible for a high volume of waste** [2]**. The WHO sees great potential in the healthcare sector to actively contribute to environmental and climate protection through more sustainable structures and processes **[3]**. The aim of this statement is to highlight the potential for optimization with regard to the appropriate use of a mouth-nose protection (medical face masks/surgical masks in accordance with DIN EN 14683** [4]**; common German abbreviation: MNS) in healthcare facilities. The challenge lies in finding the best possible balance between providing optimal hygiene standards in care for patients, including self-protection for employees, and sustainable action. **

The particle-filtering half-masks (filtering face piece; FFP; also referred to as *respiratory masks*) of protection class 2 (FFP2 mask in accordance with DIN EN 149 [5]), which are only mentioned in this statement for the sake of clarification, **serve to protect against particles, smoke, and aerosols **and are therefore to be regarded as part of personal protective equipment (PPE) [6], [7]. DIN EN 149 specifies requirements for particle-filtering half-masks used as respiratory protection, such as FFP2 masks, and also contains information on the reusability of these masks beyond the duration of a work shift [5]. In addition, there are other respiratory masks, e.g., N95 tested according to US standards and NK95 tested according to Chinese standards.

**CAVE: The indications for respiratory masks/respirators are not covered by this statement.**

The use of MNS is addressed in various recommendations by the Commission for Infection Prevention and Hygiene in Healthcare and Nursing (KRINKO; formerly the Commission for Hospital Hygiene and Infection Prevention) [6], [7]. During the Coronavirus Disease 2019 (COVID-19) pandemic, it became particularly clear how important it is to use MNS appropriately, as an estimated €3.5 billion worth of MNS were additionally imported to Germany in spring 2020 – solely due to the pandemic [8]. At the peak of the pandemic (2020 to 2022), approximately 17 billion cloth masks and surgical masks were used [9]. Worldwide, approximately 3.5 million tons of plastic waste were generated by surgical masks alone in 2020 [2]. 

With the aim of achieving infection prevention and control while at the same time acting in an environmentally friendly manner with regard to raw material consumption, production, transport and disposal of MNS, it is necessary to summarize the indications for the use of MNS in general and with regard to specific material properties, in particular for healthcare facilities. As already outlined in the KRINKO commentary on the indication-based use of disposable medical gloves in the healthcare sector [10], the appropriate use of MNS can, in the best case, lead to co-benefit solutions. 

***Life-cycle assessment of FFP2 masks vs. MNS***

According to a recent model based on a life-cycle assessment (LCA) to record the environmental impact over the entire life-cycle, the carbon dioxide footprint (CO_2_) footprint of an average FFP2/N95 mask is approximately four times higher than that of a standard multi-layer MNS: For an MNS (analogous to DIN EN 14683:2019-10 type II), the CO_2_ balance for 100 people over a period of one month is 154.33 kg CO_2_ equivalent (eq), and for N95 masks (analogous to FFP2 masks) it is 641.25 kg CO_2_eq [11]. In terms of individual products, this equates to 51.44 g CO_2_eq/MNS versus 213.75 g CO_2_eq/FFP2 mask. It should be noted that life-cycle assessments are not static figures, as many influencing factors can change over time. However, the basic statement regarding the relevant difference can be considered reliable. With regard to other environmental impacts, such as ozone depletion, acidification, eutrophication, water consumption, and resource depletion, life-cycle assessment studies comparing different types of MNS and other masks are currently lacking. However, due to the fundamentally similar production methods and material composition of the mask types, it can be assumed that N95 and FFP2 masks also have a larger carbon footprint in terms of other environmental impacts. Another ecological problem is the lack of a circular approach to the disposal and recycling of used materials, which results in high levels of fossil plastic pollution.

## 2 Definitions

In the context of infection prevention and hygiene, MNS (medical face masks/surgical masks in accordance with DIN EN 14683 [4]) primarily serve to protect others. MNS for medical use are certified as risk class 1 medical devices according to current regulations. 

Within the MNS, three types (I, II and IIR, see Table 1 (Tab. 1)) are distinguished with regard to the requirements for bacterial filter performance and spray resistance pressure, where R stands for fluid resistance (spray or splash resistance).

Liquid resistance (type IIR) is achieved through the special properties of the mask material. Information on the materials used can be found in the technical data sheets for the products. MNS masks usually consist of several layers of different polypropylene synthetic fibres. In type II or IIR surgical masks, the number and thickness of the layers is usually higher and an additional layer, e.g. made of cotton, is included. This higher use of resources and the resulting higher production and disposal costs are the main reasons for the poorer environmental performance of these surgical masks.

## 3 Infection prevention/hygiene background

MNS are used as part of a comprehensive strategy of infection prevention/hygienic measures to prevent the transmission of pathogens and reduce infections, thereby increasing patient safety. The use of MNS in medical facilities is addressed in particular in the KRINKO recommendation “Infection prevention in the care and treatment of patients with communicable diseases” [6].

**Examples of situations in which there is a general indi** **ca** **tion for wearing MNS:**

**A) For patients**

1. For self-protection: as a preventive measure against infections, e.g., in cases of immunosuppression, unless a particle-filtering half mask (FFP2 masks) is required according to the relevant risk assessment [12].
2. For the protection of others: in the event of symptoms or known infection (e.g., with influenza viruses) during contact with other persons, insofar as this is possible for the patient [6], [7].
3. For the protection of others: in the event of illness involving respiratory particle-mediated transmission (e.g., infection with respiratory syncytial viruses (RSV) or influenza viruses) during transport, insofar as this is possible/reasonable for the patient [6].
4. For the protection of others: in hospitals, in the case of known colonization with methicillin-resistant *Staphylococcus aureus* strains (MRSA) or MRSA infection in nursing and therapeutic areas when leaving the room and, if possible, for the patient during transport by the qualified emergency medical service or patient transport service [13].

**B) For employees**

1. For the protection of others: as an infection prevention/hygienic measure to protect patients from pathogen transmission

a. in certain areas, e.g., in the operating theater to prevent surgical site infections, in the pharmacy when manufacturing sterile preparations,

b. for certain particularly vulnerable patient groups, e.g., immediately after allogeneic stem cell transplantation, in cases of extensive burns,

c. during certain (aseptic) activities, e.g., insertion of a central venous catheter (CVC), joint puncture, spinal anesthesia, and

d. in certain infections of employees who are fit for work and duty, e.g., in cases of influenza-like infections or herpes labialis.

1. For self-protection: against contamination/colonisation/infection in the context of occupational safety in accordance with the risk assessment [6], [7],

a. activity-related (as protection against splashes), e.g., during open suctioning, oral care, intubation,

b. activity-related during operations where there is a potentially high risk of exposure to blood, other body fluids, secretions, excretions (e.g., laser coagulation), unless a particle-filtering half mask is required according to the risk assessment (e.g., for the removal of condylomata acuminate [14]),

c. patient-related, e.g., during direct care of MRSA-colonised/infected patients for self-protection against nasal contamination through contact [13].

1. For staff and patient protection: in the event of an epidemic or pandemic of respiratory infections, e.g., COVID-19, influenza, RSV infections, universally/non-selectively in accordance with internal or national guidelines. CAUTION: Observe occupational safety regulations for the targeted wearing of MNS or particle-filtering half masks in the event of targeted contact (section 3.B.2) [6], [7].

**C) For external parties (e.g., relatives, visitors, tradespeople)**

1. For the protection of others: contact with patients with a high predisposition for respiratory infections, e.g., immunocompromised patients or patients with their own respiratory infection, in case it is necessary (e.g., when accompanying minors).

The indications for wearing MNS should be taken into account in the local hygiene plan or book and in internal standard operating procedures (SOP) in consultation with the facility management and the responsible IPC staff and occupational health professionals, based on the KRINKO recommendations and the facility’s risk assessment, and communicated, trained, and reviewed [15], [16].

## 4 Infection prevention/hygienic measures and strategies

There are basically three approaches to reducing the ecological footprint through the use of “masks” without compromising patient safety, which are explained in more detail in sections 4.1, 4.2, and 4.3.

### 4.1 Reducing the use of MNS through strictly indication-based use 

**As a general rule, the use of a surgical mask always requires an indication **[6], [7]**, because resources can be saved by avoiding use that is not in accordance with the indications. **

**Examples of situations in which there is** **no** **fundamental indication for wearing MNS:**

**A) For patients**

- usually in cases of colonization and infection with multi-resistant pathogens (MRP). (CAVE: exception for MRSA, see section 3.A.4),
- universally outside of special epidemic situations (see section 3),
- in the case of non-communicable respiratory diseases (e.g., chronic obstructive pulmonary disease [COPD], bronchial asthma) as opposed to severe communicable respiratory infections,
- with airborne respiratory diseases outdoors at a sufficient distance,
- in single rooms.

**B) For employees**

- usually when caring for patients with MRP (CAVE: exception for MRSA, see section 3.B.2.iii),
- Universal/non-targeted for patient contact outside of specific epidemic situations (see section 3.B.3) following appropriate risk assessment.

**C) For external persons**

- universal, e.g., in the winter season,
- universal when visiting patients or patient areas.

The appropriate use of MNS requires not only the definition of the indications relevant to the local situation in the hygiene plan, but also their explanation to employees, e.g., by of training courses and setting an example, e.g., by supervisors, hygienic link personnel, and practical instructors.

In principle, the examples described in section 4.1 should be applied to the local situation, adapted to the overall hygiene plan and, if necessary, clarified. In order to achieve appropriate implementation, local situations where masks are worn without indication should be identified, understood, and avoided if possible.

### 4.2 Clarification of the areas of application for the three types of MNS defined in DIN EN 14683

**As a general rule, the “type of MNS” with the lowest hierarchical level/minimum performance requirements (see Table 1** (Tab. 1)**)** **should always be used, taking into account the applicable laws, guidelines, and internal standards.**

**According to DIN EN 14683** [4]**,** **MNS type I should only be used by patients and other persons to reduce the risk of spreading infection, particularly in epidemic or pandemic situations. MNS type I are not intended for use by medical professionals in operating theaters or other medical facilities with similar requirements. **

Not all indications for wearing a surgical mask automatically constitute indications for wearing a MNS type IIR.

For example, 

**Visitors and patients, non-medical personnel and medical professionals outside operating theaters or other medical facilities with similar requirements may use a MNS type I, **unless otherwise recommended by KRINKO in rare circumstances.

**The wearing of a MNS type II, but not a MNS type IIR, may be considered in the following situations (examples):**

- for MRSA patients to protect others,
- for employees for self-protection against contamination/colonization as part of occupational safety when caring for MRSA patients,
- for employees during procedures and operations with low exposure to splashes,
- for external personnel with respiratory pathogens for exposure prophylaxis, in the event of direct contact,
- generally for very short periods of wear, in compliance with occupational safety regulations.

**Indications for wearing a MNS type IIR are (examples):**

- activities that generate splashes (e.g., professional tooth cleaning, suctioning, oral care, intubation, surgical procedures, and operations) with expected exposure to splashes.

Occupational safety must be observed when performing aerosol-generating procedures on patients with airborne infections. For sustainability reasons, it is advisable to consider using MNS type I (CAVE: exception: according to DIN 14683 [4], MNS type I is not intended for use by medical professionals in operating theaters or other medical facilities with similar requirements) or type II instead of type IIR, and this should always be done. **The risk of confusion when using different types of MNS in the same areas for different tasks must be taken into account.**

### 4.3 Avoiding the inappropriate use of FFP2 masks

**As a general rule, the use of an FFP2 mask requires an indication, particularly in the area of occupational safety** [15]**,** [16]**. A detailed description of the indications is not the subject of this statement.**

During the COVID-19 pandemic, FFP2 masks were widely used, in some cases justifiably so from an infection prevention and control perspective. It seems at least likely (personal observations, purchasing data) that this habit has led to increased use of FFP2 masks even after the pandemic. In some internal SOPs, for example, the wearing of a surgical mask during direct contact with MRSA-colonized patients was replaced by the wearing of an FFP2 mask (personal knowledge). **Of course, there are clear infection prevention and control indications for wearing an FFP2 mask, in which case it cannot be replaced by wearing a surgical mask** (for example, see the overview table of infectious diseases and necessary measures as a basis for specifications in the hygiene plan at [7]). In contrast, during the COVID-19 pandemic, e.g., when worn collectively indoors, no clear superiority of N95 or FFP2 masks over surgical masks could be demonstrated [17], [18]. Reasons for this may be a strong dependence of the protective effect on a tight fit and a design for short-term use [17].

## 5 Outlook

Regardless of the appropriate use of these masks, there is potential for optimization in terms of environmentally conscious practices along the production and supply chains, starting with the selection of raw materials used [11] in the production processes and, in particular, logistics, with the avoidance of unnecessary transport routes. However, implementing realistic and effective measures in this area requires political decision-making processes.

## Conflict of interest

This statement was prepared on a voluntary basis and without influence from commercial interest groups on behalf of the Commission for Infection Prevention and Hygiene in Healthcare and Nursing (KRINKO) by a working group consisting of

- Prof. Dr. Simone Scheithauer (Chair),
- Prof. em. Dr. Axel Kramer,
- Prof. Dr. Christoph Lübbert,
- Prof. Dr. Nico Mutters,
- Andreas Rieß,
- Prof. Dr. Heike von Baum, and
- Prof. Dr. Julia Seifert (until May 2025).

Dr. Dieter Müller (Occupational Health Service, Goettingen University Medical Centre) and Philipp Niemeier (Team Sustainability at Procycons, Frankfurt am Main) were involved as external experts for specific issues.

Dr. Franziska Lexow, Dr. Jana Maidhof, and Marc Thanheiser from the Robert Koch Institute were also involved. 

The statement was prepared by the working group and agreed upon by the commission after extensive discussion.

## Figures and Tables

**Table 1:**
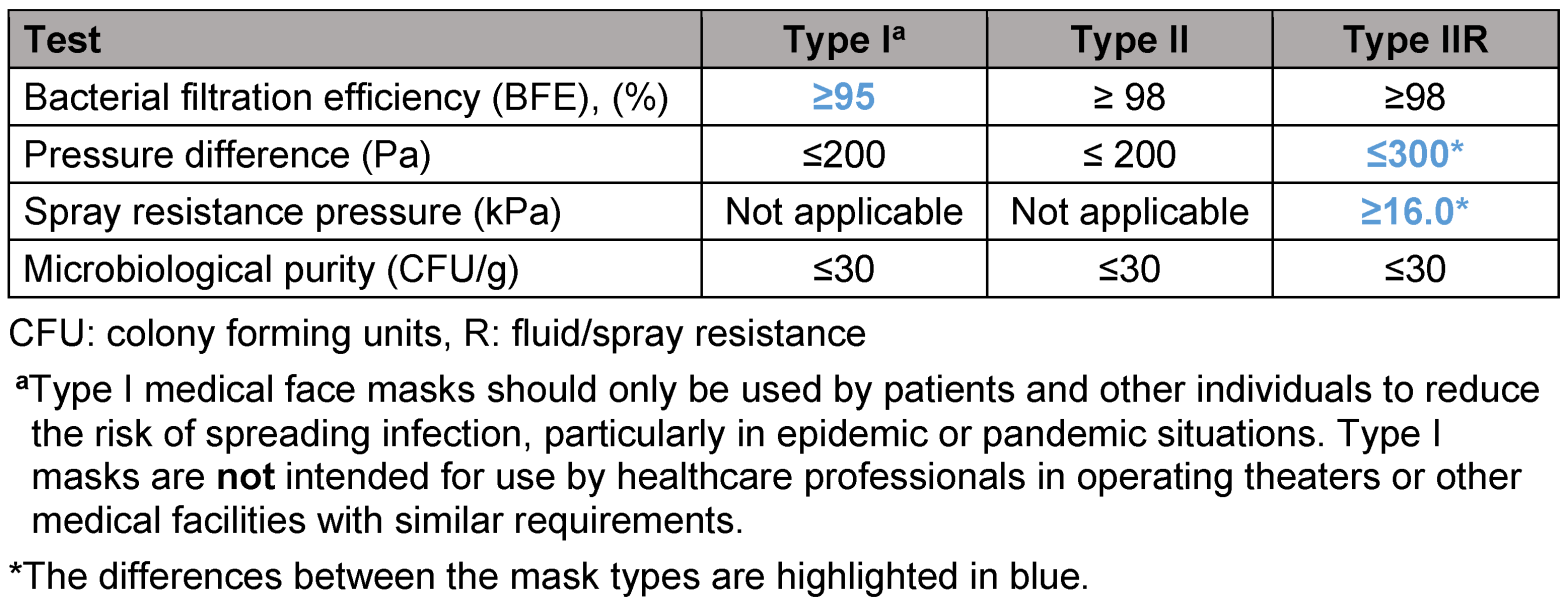
Overview of performance requirements for medical face masks according to DIN EN 14683 [4]
